# Single nucleotide polymorphisms within MUC4 are associated with colorectal cancer survival

**DOI:** 10.1371/journal.pone.0216666

**Published:** 2019-05-15

**Authors:** Shun Lu, Calogerina Catalano, Stefanie Huhn, Barbara Pardini, Linda Partu, Veronika Vymetalkova, Ludmila Vodickova, Miroslav Levy, Thomas Buchler, Kari Hemminki, Pavel Vodicka, Asta Försti

**Affiliations:** 1 Division of Molecular Genetic Epidemiology, German Cancer Research Center, Heidelberg, Germany; 2 Sichuan Cancer Hospital & Institute, Sichuan Cancer Center, School of Medicine, University of Electronic Science and Technology of China, Chengdu, China; 3 Department of Multiple Myeloma, Internal Medicine V: Hematology, Oncology and Rheumatology, Heidelberg University Hospital, Heidelberg, Germany; 4 Italian Institute for Genomic Medicine (IIGM), Turin, Italy; 5 Department of Molecular Biology of Cancer, Institute of Experimental Medicine, Academy of Sciences of the Czech Republic, Prague, Czech Republic; 6 Department of Medical Genetics, Third Faculty of Medicine, Charles University, Prague, Czech Republic; 7 Institute of Biology and Medical Genetics, 1stMedical Faculty, Charles University, Prague, Czech Republic; 8 Biomedical Centre, Faculty of Medicine in Pilsen, Charles University in Prague, Pilsen, Czech Republic; 9 Department of Oncology, Thomayer Hospital, Prague, Czech Republic; 10 Center of Primary Health Care Research, Clinical Research Center, Lund University, Malmö, Sweden; Istituto di Ricovero e Cura a Carattere Scientifico Centro di Riferimento Oncologico della Basilicata, ITALY

## Abstract

Mucins and their glycosylation have been suggested to play an important role in colorectal carcinogenesis. We examined potentially functional genetic variants in the mucin genes or genes involved in their glycosylation with respect to colorectal cancer (CRC) risk and clinical outcome. We genotyped 23 single nucleotide polymorphisms (SNPs) covering 123 SNPs through pairwise linkage disequilibrium (r^2^>0.80) in the *MUC1*, *MUC2*, *MUC4*, *MUC5AC*, *MUC6*, and *B3GNT6* genes in a hospital-based case-control study of 1532 CRC cases and 1108 healthy controls from the Czech Republic. We also analyzed these SNPs in relation to overall survival and event-free survival in a subgroup of 672 patients. Among patients without distant metastasis at the time of diagnosis, two *MUC4* SNPs, rs3107764 and rs842225, showed association with overall survival (HR 1.40, 95%CI 1.08–1.82, additive model, log-rank p = 0.004 and HR 0.64, 95%CI 0.42–0.99, recessive model, log-rank p = 0.01, respectively) and event-free survival (HR 1.31, 95%CI 1.03–1.68, log-rank p = 0.004 and HR 0.64, 95%CI 0.42–0.96, log-rank p = 0.006, respectively) after adjustment for age, sex and TNM stage. Our data suggest that genetic variation especially in the transmembrane mucin gene *MUC4* may play a role in the survival of CRC and further studies are warranted.

## Introduction

With a global incidence of 25.4/100,000 person-years, colorectal cancer (CRC) is the third most common cancer diagnosed in men and second in women [1]. Individuals with a family history of CRC have an approximately 1.87 times higher risk of developing CRC than those without a family history [2]. Lifetime risk for carriers of highly penetrant mutations in genes, such as *APC* and mismatch repair genes, may reach 50%-80% [3]. In addition, 194 low-penetrance variants located in 144 loci have so far been associated with the risk of CRC by genome-wide association studies (http://www.genome.gov/gwastudies/). Individuals carrying these variants may also have a considerable risk of CRC [4]. Early identification of such individuals could provide new options for clinical interventions and lead to cancer prevention and improved treatment [3].

Genetic variation in inflammation-related genes is an attractive research target in the context of CRC because inflammation is a known risk factor for CRC and a hallmark of human cancer in general. The gut represents a unique environment for host-pathogen interactions, with a commensal microflora in direct proximity of intestinal epithelial cells. Gut homeostasis is maintained by a physical separation of the microbial community from the gut epithelium by a mucous barrier. Secreted mucins form the physical barrier, while transmembrane mucins contribute to the protective mucous gel through their O-glycosylated tandem repeats which extend into the mucous gel [5]. The human mucin family consists of at least 22 members: MUC1*-*MUC22 [5, 6].

It has been reported that reduced synthesis and secretion of mucins and altered O-glycosylation in mucus layer are related to the causation of human ulcerative colitis [7, 8]. Dysregulation of mucin biosynthesis, especially of Muc2, and loss of core 1 and core 3-derived O-glycans have been shown to induce colitis and CRC in murine models [9–11]. Intriguingly, loss of core 3 synthase, which plays an important role in the synthesis of mucin-type O-glycans in digestive organs, has also been shown to lead to development of colon cancer in a mouse model [12].

Aberrant expression of mucins has been reported to be a common feature of CRC. MUC1 and MUC5AC have been shown to be up-regulated in CRC [13], and their overexpression has been associated with disease progression [14–20]. For MUC2, down-regulation has been associated both with CRC development and progression [14, 17, 18, 20, 21]. Aberrant expression of MUC4 during CRC progression has also been reported, and overexpression of MUC4 has been suggested to predict poor survival among patients with early stage (stage I and II) tumors but not in patients with advanced-stage (stage III and IV) tumors [19, 22], Also MUC6 has been suggested to be involved in CRC development [20, 23].

In addition to mucins, also the core 3 synthase, acetylgalactosaminyl-O-glycosyl-glycoprotein beta-1,3-N-acetylglucosaminyltransferase, encoded by the *B3GNT6* gene in humans, has been reported to be significantly down-regulated in colorectal cancer samples [24]. Due to this expression change, it was suggested to be a marker for distinguishing benign adenomas and premalignant lesions [24].

So far, few studies have investigated the association between genetic variants in the mucin genes or genes involved to their glycosylation and CRC. Here, we genotyped a set of potentially functional SNPs in the *MUC1*, *MUC2*, *MUC4*, *MUC5AC*, *MUC6*, and *B3GNT6* genes in a case-control study of 1532 CRC patients and 1108 healthy controls from the Czech Republic and evaluated their association with CRC susceptibility, progression, and prognosis.

## Materials and methods

### Study population

The case group contained 1532 CRC patients recruited between the years 2004 and 2013 in an on-going study by nine oncological departments in the Czech Republic and has been described in detail previously [25–27]. Their mean age was 63 years (range 25–91), and 61.2% of them were males. The patients showed positive colonoscopic results for malignancy, histologically confirmed as colon or rectal carcinomas. Patients who met the Amsterdam criteria I or II for hereditary nonpolyposis colorectal cancer were not included in the study. General information about gender and age at diagnosis was available for all patients. For 672 consecutively recruited, incident cases diagnosed between 2003 and 2013, clinical data at the time of diagnosis, including location of the tumor (colon/rectum) and International Union against Cancer (UICC) TNM stage classification [size or direct extent of the primary tumor (T), degree of spread to regional lymph nodes (N), presence of metastasis (M)] were available (Table 1). Also data on distant metastasis, relapse, death and last contact with the treating physician were collected.

**Table 1:** Univariable analysis of colorectal cancer survival and known prognostic factors.

Parameter	*N* ^1^	*N* ^1^^,^^2^ died (%)	HR (95%CI)	*P* value
Gender				
Female	259	99 (38.22)	1	
Male	413	204 (49.39)	**1.43 (1.13–1.82)**	**0.003**
Localisation				
Colon	429	188 (43.82)	1	
Rectum	241	113 (46.89)	1.11 (0.88–1.40)	0.39
T				
T1, T2	160	38 (23.75)	1	
T3, T4	492	247 (50.20)	**2.31 (1.64–3.25)**	**<0.0001**
N				
N0	352	100 (28.41)	1	
N1, N2	285	173 (60.70)	**2.66 (2.08–3.41)**	**<0.0001**
M				
M0	494	157 (31.78)	1	
M1	178	146 (82.02)	**3.90 (3.09–4.91)**	**<0.0001**
TNM Stage				
Stage I	122	25 (20.94)	1	
Stage II	195	49 (25.13)	1.07 (0.66–1.73)	0.8
Stage III	177	83 (46.89)	**2.26 (1.44–3.53)**	**0.0004**
Stage IV	178	146 (82.02)	**5.70 (3.72–8.74)**	**< .0001**

^1^Number of cases may differ due to missing data

^2^Includes only individuals who died during the follow-up time

*N*, number of patients; HR, hazard ratio; CI, confidence interval. Bold numbers indicate a statistical significance at 5% level

The control group consisted of 1108 healthy individuals recruited by a blood-donor center in one hospital in Prague. These disease-free individuals represent the general population of the Czech Republic, which has a genetically quite uniform population [28]. Their mean age was 47 years (range 18–94) and 53.3% of them were males. All participants were of Czech Caucasian origin

All procedures performed involving human participants were in accordance with the ethical standards of the institutional and/or national research committee and with the 1964 Helsinki declaration and its later amendments or comparable ethical standards. The study was approved by the ethical committees of the participating institutes, the Institute of Experimental Medicine, Academy of Sciences of the Czech Republic, the Institute of Clinical and Experimental Medicine and Faculty Thomayer Hospital, and the General University Hospital, all in Prague, Czech Republic. Written informed consent was obtained from all individual participants included in this study.

### SNP selection and functional prediction of the associated variants

Five first and most studied mucin genes *MUC1*, *2*, *4*, *5AC* and *6* and the gene encoding the core 3 synthase, *B3GNT6*, which plays an important role in the synthesis of mucin-type O-glycans in digestive organs, were selected as candidate genes based on their functional role in CRC development and progression. A total of 23 SNPs were selected in these genes from the International HapMap Project (http://hapmap.ncbi.nlm.nih.gov) and the NCBI database (http://www.ncbi.nlm.nih.gov) (Table 2) based on the criteria described in[26]: location within the coding region (non-synonymous SNPs), the 3’ and 5’ untranslated regions (UTRs) and the promoter (up to approximately 1 kb from the transcription start site), pairwise linkage disequilibrium (LD, r^2^≤0.80) between the SNPs in Utah residents with Northern and Western European ancestry from the CEPH collection (CEU). We selected only SNPs with the minor allele frequency (MAF) ≥ 10% in Europeans, to minimize statistical uncertainties in the survival analysis. The selected SNPs provided information on altogether 123 SNPs due to LD (r^2^>0.80). For the SNPs, which associated with CRC risk or survival, SNPnexus (http://snp-nexus.org/) was used to predict functional consequences of the selected SNPs. We also used additional web-based tools [HaploReg v2 (http://www.broadinstitute.org), Regulome DB (http://regulome.stanford.edu/) and SNPinfo Web Server (http://snpinfo.niehs.nih.gov/cgi-bin/snpinfo/snpfunc.cgi)] to predict their effects on potential regulatory elements.

**Table 2:** Polymorphisms evaluated in this study.

dbSNP rs#	Alleles	Gene	Chromosome	Location	MAF^1^	SNP captured with r2 ≥ 0.80^2^
rs12743084	G/C	MUC1	1q22	Exon	0.415	
rs4072037	A/G	MUC1	1q22	Exon	0.37	rs12411216 rs2974937 rs370545 rs914615
						rs2066981 rs11355526 rs2075570 rs28445596
						rs2990220 rs497829 rs2049805 rs2974931
						rs2974930 rs2974929 rs2990245 rs2974935
rs11825977	A/G	MUC2	11p15.5	Exon	0.226	
rs2071175	C/T	MUC2	11p15.5	Promotor	0.1	
rs2856111	C/T	MUC2	11p15.5	Exon	0.105	
rs3749331	C/T	MUC4	3q29	Exon	0.24	rs62284986 rs62282501 rs60632417
rs3107764	C/G	MUC4	3q29	Exon	0.4	
rs2246901	A/C	MUC4	3q29	Exon	0.167	
rs842225	A/G	MUC4	3q29	5' UTR	0.48	
rs35783651	G/C	MUC5ac	11p15.5	Exon	0.15	rs28513455 rs28699476 rs34974357 rs28653192
						rs35705491 rs34462515 rs35779873 rs34831688
						rs35288961 rs35525357 rs35968147 rs28728088
						rs3087562 rs13010 rs11347 rs13380
						rs28562881 rs28666868 rs28429038 rs28504415
						rs28414902 rs150936581 rs35915689 rs141032511
						rs35700114
rs17859812	G/A	MUC5ac	11p15.5	5' UTR	0.314	rs34664315 rs36021067 rs34207169 rs35396393
						rs28691231 rs28542750 rs28737416 rs28434250
						rs28524833 rs28645549 rs28639518 rs28468624
						rs28569104 rs28520914 rs28633709 rs28663568
						rs28464760 rs28972401 rs28550725 rs28653550
						rs2075841 rs55898663 rs2075843 rs28457780 rs28439383 rs28520579 rs28519516 rs28545782 rs28558973 rs28368633 rs28731161 rs28399941 rs28414902 rs150936581 rs35915689 rs141032511 rs35700114
rs11604757	C/T	MUC6	11p15.5	Exon	0.139	
rs61869016	G/A	MUC6	11p15.5	5' UTR	0.39	
rs6597947	A/C	MUC6	11p15.5	5' UTR	0.117	rs6597946
rs72842418	T/C	MUC6	11p15.5	5' UTR	0.131	
rs7396383	A/T	MUC6	11p15.5	Exon	0.2	
rs7481521	C/T	MUC6	11p15.5	Exon	0.434	rs12276666 rs12281858
rs12271271	G/A	B3GNT6	11p15.5	5' UTR	0.308	rs11237061
rs12422079	A/C	B3GNT6	11p15.5	5' UTR	0.293	rs34153015
rs58116088	G/A	B3GNT6	11p15.5	3' UTR	0.317	rs7115080 rs72949248 rs12575731 rs7103667
rs61902094	G/A	B3GNT6	11p15.5	3' UTR	0.142	rs77887719 rs61902097 rs61902099 rs11600516
						rs58520141 rs61902104 rs11603853
rs6592699	A/G	B3GNT6	11p15.5	5' UTR	0.256	rs6592698 rs6592700 rs12292060 rs1894008
rs73493606	T/C	B3GNT6	11p15.5	Exon	0.18	rs60414780 rs77669632 rs78494560 rs79335393
						rs11600807 rs7110184 rs76695415 rs11605987
						rs11237071 rs60341963 rs12274379 rs74567524
						rs2186657 rs75007589 rs56839740 rs58336054
						rs12284354 rs12270821 rs11237077 rs12291669 rs112674340 rs56937577

^1^Minor allele frequency (MAF) based on Utah residents with Northern and Western European ancestry from the CEPH collection in the HapMap project

^2^Pairwise linkage disequilibrium (r^2^) was calculated for the SNPs with MAF ≥ 10% within the regions of interest based on Utah residents with Northern and Western European ancestry from the CEPH collection in the HapMap project

### Genotyping

As described in detail in our previous study, whole genome amplified (WGA) DNA from peripheral blood leukocytes was used [29]. All WGA samples were genotyped for two common SNPs: less than 0.1% of the genotypes could not be determined or they did not agree with the corresponding genomic DNA sample, confirming the accuracy of WGA. KASP (LGC Genomics) or TaqMan (Applied Biosystems) allelic discrimination methods were used to genotype the selected SNPs. DNA amplification was performed according to the LGC Genomics’ and TaqMan´s PCR conditions. Case and control samples were amplified simultaneously in 384-well format using Hydrocycler 16 (LGC Genomics). Endpoint genotype detection was carried out on the ViiA 7 Real-Time PCR System (Applied Biosystems). The sample set contained 142 duplicated samples as quality controls. The genotype correlation between the duplicate samples was > 99%.

### Statistical analysis

The observed genotype frequencies in the controls were tested for Hardy–Weinberg equilibrium (HWE) using the chi-square test. Odds ratios (ORs) and 95% confidence intervals (CIs) for associations between genotypes and CRC risk were calculated by logistic regression (PROC LOGISTIC, SAS Version 9.3; SAS Institute, Cary, NC), and adjusted for age and gender. Co-dominant, dominant and recessive models were calculated to evaluate the statistical significance. The major allele homozygous genotype was used as the reference. In the case of recessive model, a combination of major allele homozygous and heterozygous genotypes was used as the reference. To account for multiple testing, the SNP Spectral Deposition (SNPSpD) method for multilocus analyses was applied. For a polymorphism with a variant allele frequency between 10 and 50%, the study had greater than 90% power to detect an OR of 1.50 at a significance level of 0.05 (PS—software for power and sample size calculation, http://biostat.mc.vanderbilt.edu/twiki/bin/view/Main/PowerSampleSize). In this study, we analyzed overall survival both in the 672 CRC patients diagnosed between 2003 and 2013 and in a subgroup of 494 patients with non-metastatic disease at the time of diagnosis, using the date of death or last contact with the treating physician as the end point of follow-up. Median follow-up time for the 672 patients was 58 months, while for patients who did not have distant metastasis at the time of diagnosis it was 65 months. For event-free survival in patients with non-metastatic disease at the time of diagnosis, date of distant metastasis, relapse, death or last contact with the treating physician was used as the end point of follow-up. Median follow-up time was 55 months. Survival curves for overall and event-free survival were derived by the Kaplan–Meier method (PROC LIFETEST, SAS Version 9.3) and compared using log-rank test. The relative risk of death was estimated as hazard ratio (HR) using Cox regression (PROC PHREG, SAS Version 9.2). Multivariable survival analyses were adjusted for age, gender, T, N, M, TNM stage and grade separately, and in a final model for age, sex, and TNM stage. Proportional hazards assumption was tested and the assumption was fulfilled for all SNPs.

## Results

Altogether, 123 SNPs with MAF ≥ 10% in the CEU population were located within the regions of interest (promoter, 5’- and 3’-UTR, non-synonymous SNPs) of the 6 genes *MUC (1*, *2*, *4*, *5AC*, *6)* and *B3GNT6*. From these, 23 SNPs were selected for genotyping based on LD (r^2^≤0.80) (Table 2). The genotype distribution of all genotyped polymorphisms was consistent with HWE in the control group (*P*>0.05), except for rs72842418 (*MUC6*), which was excluded from the analyses. The MAFs in the control population were similar to the ones reported by the HapMap project for the CEU population.

Minor allele carriers of the *MUC6* 5’UTR SNP rs61869016 had a decreased risk of CRC (OR 0.78, 95%CI 0.64–0.96, p = 0.02) (S1 Table). To correct for multiple testing, we used the SNPSpD approach. The study-wise effective number of independent markers Meff was calculated to be 18, which gave the significance threshold of 0.0028. Thus, the association with the SNP rs61869016 (*MUC6*) did not remain formally significant (P = 0.02).

**Fig 1:**
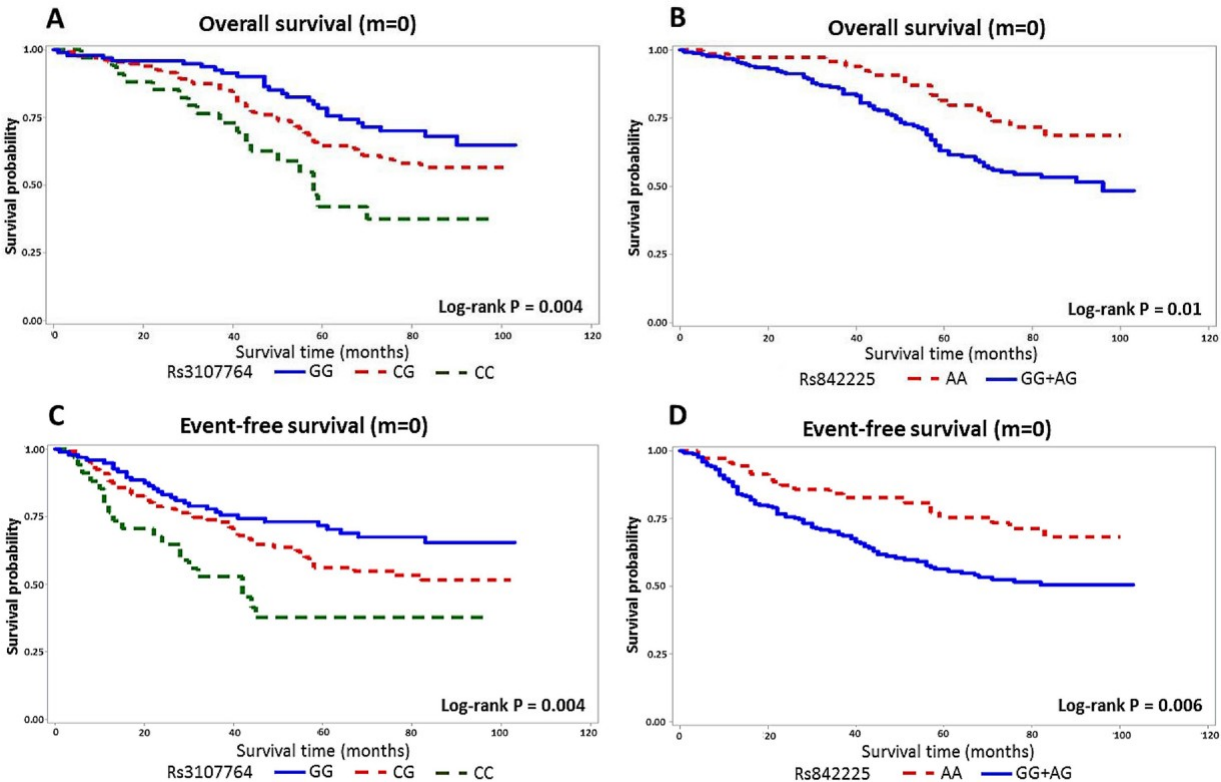
Kaplan-Meier analysis of survival among colorectal cancer patients without distant metastasis at diagnosis (n = 494) according to genotypes of *MUC4* rs3107764 and rs842225. (A) rs3107764: overall survival; (B) rs3107764: event-free survival; (C) rs842225: overall survival; (D) rs842225: event-free survival.

In the univariable analysis, the following parameters were associated with overall survival rate: gender, T, N, M and TNM stage (Table 1). Three SNPs, rs58116088 in *B3GNT6* and rs2071175 and rs2856111 in *MUC2*, showed nominal associations with overall survival among the 672 patients with follow-up data after adjustment for age, sex and TNM stage (HR 0.78, 95%CI 0.62–0.99, dominant model, HR 4.47, 95%CI 1.09–18.30 for TT homozygotes, HR 2.55, 95%CI 1.25–5.21 for CC homozygotes, respectively, S2 Table). Interestingly, among patients without distant metastasis at the time of diagnosis two *MUC4* SNPs, rs3107764 and rs842225, showed associations with overall survival (HR 1.40, 95%CI 1.08–1.82, additive model and HR 0.64, 95%CI 0.42–0.99, recessive model, respectively) and event-free survival (HR 1.31, 95%CI 1.03–1.68 and HR 0.64, 95%CI 0.42–0.96, respectively) after adjustment for age, sex and TNM stage (Table 3). The Kaplan-Meier survival curves representing survival rates of the patients according to their rs3107764 and rs842225 genotypes are presented in Fig 1. The overall and event-free survival differences of the patients without distant metastasis at the time of diagnosis between the carriers of the different genotypes were statistically significant for rs3107764 in the 3-genotype model with log-rank p-values of 0.004 (overall survival) and 0.004 (event-free survival), respectively, and for rs842225 in recessive model with log-rank p-values of 0.01 and 0.006, respectively.

**Table 3:** Associations of rs2071175, rs58116088, rs3107764 and rs842225 with overall survival of all patients and overall and event-free survival among patients without distant metastasis at the time of diagnosis (M = 0).

Gene	SNP ID	Genotype	Overall Survival Analysis^1^ (adjusted for age, sex and stage)				Overall Survival Analysis (M = 0)^2^ adjusted for age, sex and stage				Event-free Survival Analysis (M = 0) ^3^ adjusted for age, sex and stage			*P* value
			N^4^	N^4^^,^^5^ died (%)	HR	*P* value	N^4^	N^4^^,^^5^ died (%)	HR	*P* value	N^4^	N^4^^,^^5^ died (%)	HR	
MUC2	rs2071175	C/C	513	221 (43.08)	Ref.		382	115 (30.10)	Ref.		382	127 (33.25)	1	
		C/T	38	20 (52.63)	0.94 (0.59–1.50)	0.81	26	11 (42.31)	1.47 (0.79–2.76)	0.23	26	11 (42.31)	1.17 (0.63–2.17)	0.63
		T/T	4	2 (50.00)	**4.47 (1.09–18.30)**	**0.04**	3	1 (33.33)	4.14 (0.56–30.46)	0.16	3	1 (33.33)	2.46 (0.34–17.91)	0.37
	Dominant model	C/T+ T/T	42	22 (52.38)	1.02 (0.66–1.59)	0.93	29	12(41.38)	1.56 (0.85–2.85)	0.15	29	12 (41.38)	1.22 (0.67–2.22)	0.51
B3GNT6	rs58116088	G/G	239	115 (48.12)	Ref.		172	58 (33.72)	Ref.		172	65 (37.79)	1	
		G/A	318	130 (40.88)	0.79 (0.62–1.02)	0.07	242	72 (29.75)	0.77 (0.54–1.09)	0.14	242	78(32.23)	0.78 (0.56–1.09)	0.14
		A/A	87	42 (48.28)	0.75 (0.53–1.07)	0.11	61	19 (31.15)	0.75 (0.45–1.27)	0.29	61	22 (36.07)	0.87 (0.54–1.41)	0.57
	Dominant model	G/A+A/A	405	172 (42.47)	**0.78 (0.62–0.99)**	**0.04**	303	91 (30.03)	0.76 (0.55–1.07)	0.11	303	100 (33.00)	0.80 (0.58–1.09)	0.16
MUC4	rs3107764	G/G	199	84 (42.21)	Ref.		152	40 (26.32)	Ref.		152	45 (29.61)	1	
		C/G	283	119 (42.05)	0.97 (0.73–1.29)	0.84	215	65 (30.23)	1.13 (0.76–1.69)	0.54	215	75 (34.88)	1.14 (0.79–1.66)	0.48
		C/C	86	40 (46.51)	1.06 (0.72–1.55)	0.77	66	26 (39.39)	2.08 (1.26–3.43)	0.004	66	28 (42.42)	1.79 (1.12–2.88)	0.016
	Additive model		568	243 (42.78)	1.02 (0.84–1.23)	0.85	433	131 (30.25)	**1.40 (1.08–1.82)**	**0.01**	433	148 (34.18)	**1.31 (1.03–1.68)**	**0.03**
MUC4	rs842225	G/G	176	79 (44.89)	Ref.		135	45 (33.33)	Ref.		135	50 (37.04)	1	
		A/G	295	131 (44.41)	1.06 (0.80–1.40)	0.7	216	69 (31.94)	0.92 (0.63–1.34)	0.66	216	77 (35.65)	0.96 (0.68–1.38)	0.84
		A/A	144	63 (43.75)	0.96 (0.69–1.34)	0.83	103	26 (25.24)	0.61 (0.37–0.99)	0.045	103	28 (27.18)	0.62 (0.39–0.99)	0.047
		G/G+A/G	471	210 (44.59)	Ref.		351	114 (32.48)	Ref.		351	127 (36.18)	1	
	Recessive model	A/A	144	63 (43.75)	0.93 (0.70–1.23)	0.62	103	26 (25.24)	**0.64 (0.42–0.99)**	**0.04**	103	28 (27.18)	**0.64 (0.42–0.96)**	**0.03**

^1^Overall survival was calculated for all patients diagnosed between 2003 and 2013 (n = 672)

^2^Overall survival was calculated for patients diagnosed between 2003 and 2013, who did not have distant metastasis at the time of diagnosis (n = 494)

^3^Event-free survival was calculated for patients diagnosed between 2003 and 2013, who did not have distant metastasis at the time of diagnosis (n = 494)

^4^Number of cases may differ due to missing data.

^5^Includes only individuals who died during the follow-up time.

N, number of patients; OR, odds ratio; CI, confidence interval; Bold numbers indicate a statistical significance at 5% level; Ref, reference genotype(s)

## Discussion

Several mucin-type O-glycans have been considered as prognostic markers in CRC due to their aberrant expression [15, 18, 20, 22, 24]. Recently, their interaction with Wnt/β-catenin signaling has been shown to play an important role in CRC progression [30]. In this genetic association study, we investigated the associations between 23 SNPs capturing 123 potentially functional SNPs in the mucin genes and *B3GNT6* and CRC risk and clinical outcome. SNP rs61869016 located in the *MUC6* gene exhibited a nominal association with CRC risk. In the multivariable survival analysis, two SNPs located in *MUC4* were associated with overall and event-free survival of non-metastatic CRC patients.

MUC6 has recently been proposed as a prognostic marker for CRC patients [23]. Although MUC6 expression was rarely observed in colorectal tumors, when it was present, the patients had a very good prognosis [23]. In another study, MUC6 expression was associated with the CpG island methylator phenotype (CIMP) of CRC and tumorigenesis via the serrated neoplasia pathway [31]. So far, most studies on polymorphisms in *MUC6* have been dedicated to gastric cancer, without any significant associations [32]. The only study on CRC reported no association between a 3’UTR SNP and risk or clinical outcome [25]. In our study, this SNP was captured by rs7481521 (r^2^ = 0.72) and similar to the previous study no association was observed. However, we observed a nominally decreased risk among carriers of the minor allele of rs61869016 in the 5' UTR of *MUC6*. According to Regulome DB the SNP is located in a region showing weak repressed Polycomb marks in different colorectal tissues, while in gastric tissue, it shows enhancer marks. Thus, a potential mechanism how rs61869016 could affect CRC risk is through tissue-specific regulation of gene expression via changes in histone state, which may be relevant for the development of some specific types of CRC.

Previous reports have indicated that MUC4 expression is lost as CRC progresses, and this loss of MUC4 may be regulated by β-catenin [33], however, in a subgroup of patients with overexpression of MUC4 a worse prognosis has been reported [22, 34]. Through its epidermal growth factor (EGF) domains, MUC4 may act as an intramembrane ligand for receptor tyrosine kinase ErbB2 and execute antiapoptotic function and by that promote tumor progression [35, 36]. Published studies have reported that polymorphisms in *MUC4* are associated with susceptibility to lung cancer, endometriosis development and endometriosis-related infertility [37, 38]; homozygous G allele carriers of the *MUC4* SNP rs842225 were reported to have a decreased risk of lung cancer in a Han Chinese population [35, 37, 38]. So far, no studies have investigated the relationship between *MUC4* SNPs and CRC. In our study, we found two SNPs in *MUC4*, rs3107764 and rs842225, to be associated with overall and event-free survival among patients without distant metastasis at the time of diagnosis. C allele carriers of rs3107764 had an increased risk of dying while homozygous A allele carriers of rs842225 had a decreased risk. Rs842225 is predicted to be likely to affect binding and expression of a target gene (*MUC20*) by Regulome DB. Chromatin state data indicated also marks of strong transcription at this locus in colon and rectal tissues. Rs3107764, on the other hand, is a missense SNP (Ala41Pro), which is predicted to be possibly damaging by PolyPhen. It may also affect expression of *MUC20*, although with a lower extent than rs842225. Previously, overexpression of MUC20 has been associated with recurrence and poor survival of CRC patients [39].

Given the important role of the mucous barrier in maintaining the gut homeostasis, it is surprising that only four studies have been published regarding potential associations between genetic variants in the mucin genes and CRC [25, 40–42]. Similarly, studies on polymorphisms in mucin genes or on alterations in mucin expression affecting risk of inflammatory bowel diseases are sparse and inconclusive[43]. The four studies on SNPs and CRC risk reported either no or only weak associations[25, 38–40], similar to our study. However, the study investigating also associations with clinical outcome reported three associations between SNPs located in the microRNA binding sites, recurrence and survival [25]. Interestingly, the genes involved were *MUC17*, *MUC20* and *MUC21*, all encoding transmembrane mucins, similar to *MUC4*, which was identified in our study to affect both overall and event-free survival in metastasis-free patients at the time of diagnosis. In addition to protecting gut epithelia against micro-organisms and inflammation, transmembrane mucins also play an important role in transmitting cell-cell and cell-microenvironment signals [5, 44]. In addition, they can induce cell transformation and promote tumor progression [5, 44]. Thus, genetic variation in the regulatory regions of the transmembrane mucin genes may modify the function of the corresponding proteins and by that affect colorectal tumor progression and survival of the patients.

Our study has both strengths and limitations. The cases and controls represent a genetically quite uniform Czech population [28], excluding the problem of population stratification. The control group consisted of healthy blood donors, who may be more health conscious than the general population. The control group was also younger and the proportion of men was lower than in the case group. To avoid bias due to these differences, we adjusted our analyses for both age and sex. From the 1532 CRC patients included in the study, 672 consecutively collected, incident cases diagnosed between 2003 and 2013 were available for the survival analysis. This ensured that only newly diagnosed CRC cases (within 1 year of diagnosis before enrollment for this study) were included in the study, excluding a survival bias. For this subgroup, nearly complete clinical data were available, allowing evaluation of the SNPs as independent prognostic markers. However, although the number of individuals for the risk analysis was sufficient for this kind of a study, the limitation to newly diagnosed CRC cases in the survival analyses decreased the power to detect associations with genotypes. Because of that we concentrated on SNPs with MAF ≥ 10% in Europeans. Although we covered a total of 123 SNPs by the 23 genotyped SNPs in the basic regulatory and coding regions of the genes, it is possible that SNPs with lower MAF or SNPs in still unknown regulatory regions of these genes, such as the enhancer and the silencer regions, might also have an effect on CRC susceptibility or clinical outcome. As our study covered only 6 of the known mucin and mucin-type O-glycosylation genes, further studies are warranted in the other genes of this system maintaining the gut homeostasis.

In summary, our results on associations of SNPs in *MUC4* with survival of CRC patients supports previous studies implicating importance of genetic variants in mucin genes encoding transmembrane mucins in the clinical outcome of CRC patients. Further studies with larger independent populations are needed to verify our findings and to investigate the potential function of the studied SNPs as well as SNPs in other relevant regulatory regions of the mucin-type O-glycans.

## Supporting information

S1 Table Association of all evaluated SNPs with colorectal cancer susceptibility in the study population of 1532 cases and 1108 controls. (DOCX)

S2 Table Association of all evaluated SNPs with overall survival of all colorectal cancer patients (n = 672) and overall and event-free survival among patients without distant metastasis at the time of diagnosis (M = 0, n = 494). (DOCX)

## References

[1] Global Burden of Disease Cancer C, Fitzmaurice C, Allen C, Barber RM, Barregard L, Bhutta ZA, et al Global, Regional, and National Cancer Incidence, Mortality, Years of Life Lost, Years Lived With Disability, and Disability-Adjusted Life-years for 32 Cancer Groups, 1990 to 2015: A Systematic Analysis for the Global Burden of Disease Study. JAMA oncology. 2017;3(4):524–48. 10.1001/jamaoncol.2016.5688 . 27918777 PMC6103527

[2] Frank C, Fallah M, Ji J, Sundquist J, Hemminki K. The population impact of familial cancer, a major cause of cancer. International journal of cancer. 2014;134(8):1899–906. 10.1002/ijc.28510 . 24590453

[3] Stoffel EM, Yurgelun MB. Genetic predisposition to colorectal cancer: Implications for treatment and prevention. Seminars in oncology. 2016;43(5):536–42. 10.1053/j.seminoncol.2016.08.002 . 27899184

[4] Sud A, Kinnersley B, Houlston RS. Genome-wide association studies of cancer: current insights and future perspectives. Nat Rev Cancer. 2017;17(11):692–704. 10.1038/nrc.2017.82 . 29026206

[5] Kufe DW. Mucins in cancer: function, prognosis and therapy. Nature reviews Cancer. 2009;9(12):874–85. 10.1038/nrc2761 19935676 PMC2951677

[6] Hijikata M, Matsushita I, Tanaka G, Tsuchiya T, Ito H, Tokunaga K, et al Molecular cloning of two novel mucin-like genes in the disease-susceptibility locus for diffuse panbronchiolitis. Human genetics. 2011;129(2):117–28. 10.1007/s00439-010-0906-4 . 20981447

[7] Johansson ME, Sjovall H, Hansson GC. The gastrointestinal mucus system in health and disease. Nature reviews Gastroenterology & hepatology. 2013;10(6):352–61. 10.1038/nrgastro.2013.35 23478383 PMC3758667

[8] Bergstrom KS, Xia L. Mucin-type O-glycans and their roles in intestinal homeostasis. Glycobiology. 2013;23(9):1026–37. 10.1093/glycob/cwt045 23752712 PMC3858029

[9] Velcich A, Yang W, Heyer J, Fragale A, Nicholas C, Viani S, et al Colorectal cancer in mice genetically deficient in the mucin Muc2. Science. 2002;295(5560):1726–9. 10.1126/science.1069094 . 11872843

[10] Van der Sluis M, De Koning BA, De Bruijn AC, Velcich A, Meijerink JP, Van Goudoever JB, et al Muc2-deficient mice spontaneously develop colitis, indicating that MUC2 is critical for colonic protection. Gastroenterology. 2006;131(1):117–29. 10.1053/j.gastro.2006.04.020 . 16831596

[11] Fu J, Wei B, Wen T, Johansson ME, Liu X, Bradford E, et al Loss of intestinal core 1-derived O-glycans causes spontaneous colitis in mice. The Journal of clinical investigation. 2011;121(4):1657–66. 10.1172/JCI45538 21383503 PMC3069788

[12] An G, Wei B, Xia B, McDaniel JM, Ju T, Cummings RD, et al Increased susceptibility to colitis and colorectal tumors in mice lacking core 3-derived O-glycans. The Journal of experimental medicine. 2007;204(6):1417–29. 10.1084/jem.20061929 17517967 PMC2118614

[13] Zeng Y, Zhang Q, Zhang Y, Lu M, Liu Y, Zheng T, et al MUC1 Predicts Colorectal Cancer Metastasis: A Systematic Review and Meta-Analysis of Case Controlled Studies. PloS one. 2015;10(9):e0138049 10.1371/journal.pone.0138049 26367866 PMC4569423

[14] Kesari MV, Gaopande VL, Joshi AR, Babanagare SV, Gogate BP, Khadilkar AV. Immunohistochemical study of MUC1, MUC2 and MUC5AC in colorectal carcinoma and review of literature. Indian journal of gastroenterology: official journal of the Indian Society of Gastroenterology. 2015;34(1):63–7. 10.1007/s12664-015-0534-y . 25731647

[15] Kocer B, Soran A, Erdogan S, Karabeyoglu M, Yildirim O, Eroglu A, et al Expression of MUC5AC in colorectal carcinoma and relationship with prognosis. Pathology international. 2002;52(7):470–7. . 12167106 10.1046/j.1440-1827.2002.01369.x

[16] Renaud F, Mariette C, Vincent A, Wacrenier A, Maunoury V, Leclerc J, et al The serrated neoplasia pathway of colorectal tumors: Identification of MUC5AC hypomethylation as an early marker of polyps with malignant potential. International journal of cancer. 2016;138(6):1472–81. 10.1002/ijc.29891 . 26476272

[17] Renaud F, Vincent A, Mariette C, Crepin M, Stechly L, Truant S, et al MUC5AC hypomethylation is a predictor of microsatellite instability independently of clinical factors associated with colorectal cancer. International journal of cancer. 2015;136(12):2811–21. 10.1002/ijc.29342 . 25403854

[18] Imai Y, Yamagishi H, Fukuda K, Ono Y, Inoue T, Ueda Y. Differential mucin phenotypes and their significance in a variation of colorectal carcinoma. World journal of gastroenterology. 2013;19(25):3957–68. 10.3748/wjg.v19.i25.3957 23840140 PMC3703182

[19] Biemer-Huttmann AE, Walsh MD, McGuckin MA, Ajioka Y, Watanabe H, Leggett BA, et al Immunohistochemical staining patterns of MUC1, MUC2, MUC4, and MUC5AC mucins in hyperplastic polyps, serrated adenomas, and traditional adenomas of the colorectum. The journal of histochemistry and cytochemistry: official journal of the Histochemistry Society. 1999;47(8):1039–48. 10.1177/002215549904700808 . 10424888

[20] Molaei M, Mansoori BK, Mashayekhi R, Vahedi M, Pourhoseingholi MA, Fatemi SR, et al Mucins in neoplastic spectrum of colorectal polyps: can they provide predictions? BMC cancer. 2010;10:537 10.1186/1471-2407-10-537 20929551 PMC2958948

[21] Ogata S, Uehara H, Chen A, Itzkowitz SH. Mucin gene expression in colonic tissues and cell lines. Cancer research. 1992;52(21):5971–8. . 1394223

[22] Shanmugam C, Jhala NC, Katkoori VR, Wan W, Meleth S, Grizzle WE, et al Prognostic value of mucin 4 expression in colorectal adenocarcinomas. Cancer. 2010;116(15):3577–86. 10.1002/cncr.25095 20564074 PMC2910814

[23] Betge J, Schneider NI, Harbaum L, Pollheimer MJ, Lindtner RA, Kornprat P, et al MUC1, MUC2, MUC5AC, and MUC6 in colorectal cancer: expression profiles and clinical significance. Virchows Archiv: an international journal of pathology. 2016;469(3):255–65. 10.1007/s00428-016-1970-5 27298226 PMC5007278

[24] Iwai T, Kudo T, Kawamoto R, Kubota T, Togayachi A, Hiruma T, et al Core 3 synthase is down-regulated in colon carcinoma and profoundly suppresses the metastatic potential of carcinoma cells. Proc Natl Acad Sci U S A. 2005;102(12):4572–7. 10.1073/pnas.0407983102 15755813 PMC555466

[25] Vymetalkova V, Pardini B, Rosa F, Jiraskova K, Di Gaetano C, Bendova P, et al Polymorphisms in microRNA binding sites of mucin genes as predictors of clinical outcome in colorectal cancer patients. Carcinogenesis. 2017;38(1):28–39. 10.1093/carcin/bgw114 . 27803053

[26] Lu S, Pardini B, Cheng B, Naccarati A, Huhn S, Vymetalkova V, et al Single nucleotide polymorphisms within interferon signaling pathway genes are associated with colorectal cancer susceptibility and survival. PLoS One. 2014;9(10):e111061 10.1371/journal.pone.0111061 25350395 PMC4211713

[27] Huhn S, Bevier M, Pardini B, Naccarati A, Vodickova L, Novotny J, et al Colorectal cancer risk and patients' survival: influence of polymorphisms in genes somatically mutated in colorectal tumors. Cancer Causes Control. 2014;25(6):759–69. 10.1007/s10552-014-0379-1 . 24706189

[28] Nelis M, Esko T, Magi R, Zimprich F, Zimprich A, Toncheva D, et al Genetic structure of Europeans: a view from the North-East. PLoS One. 2009;4(5):e5472 10.1371/journal.pone.0005472 19424496 PMC2675054

[29] Lu S, Bevier M, Huhn S, Sainz J, Lascorz J, Pardini B, et al Genetic variants in C-type lectin genes are associated with colorectal cancer susceptibility and clinical outcome. Int J Cancer. 2013;133(10):2325–33. 10.1002/ijc.28251 . 23650115

[30] Pai P, Rachagani S, Dhawan P, Batra SK. Mucins and Wnt/beta-catenin signaling in gastrointestinal cancers: an unholy nexus. Carcinogenesis. 2016;37(3):223–32. 10.1093/carcin/bgw005 26762229 PMC5014091

[31] Walsh MD, Clendenning M, Williamson E, Pearson SA, Walters RJ, Nagler B, et al Expression of MUC2, MUC5AC, MUC5B, and MUC6 mucins in colorectal cancers and their association with the CpG island methylator phenotype. Modern pathology: an official journal of the United States and Canadian Academy of Pathology, Inc. 2013;26(12):1642–56. 10.1038/modpathol.2013.101 . 23807779

[32] Wen R, Gao F, Zhou CJ, Jia YB. Polymorphisms in mucin genes in the development of gastric cancer. World journal of gastrointestinal oncology. 2015;7(11):328–37. 10.4251/wjgo.v7.i11.328 26600932 PMC4644855

[33] Pai P, Rachagani S, Dhawan P, Sheinin YM, Macha MA, Qazi AK, et al MUC4 is negatively regulated through the Wnt/beta-catenin pathway via the Notch effector Hath1 in colorectal cancer. Genes & cancer. 2016;7(5–6):154–68. 10.18632/genesandcancer.108 27551331 PMC4979589

[34] Huang X, Wang X, Lu SM, Chen C, Wang J, Zheng YY, et al Clinicopathological and prognostic significance of MUC4 expression in cancers: evidence from meta-analysis. International journal of clinical and experimental medicine. 2015;8(7):10274–83. 26379819 PMC4565202

[35] Jepson S, Komatsu M, Haq B, Arango ME, Huang D, Carraway CA, et al Muc4/sialomucin complex, the intramembrane ErbB2 ligand, induces specific phosphorylation of ErbB2 and enhances expression of p27(kip), but does not activate mitogen-activated kinase or protein kinaseB/Akt pathways. Oncogene. 2002;21(49):7524–32. 10.1038/sj.onc.1205970 . 12386815

[36] Jonckheere N, Skrypek N, Frenois F, Van Seuningen I. Membrane-bound mucin modular domains: from structure to function. Biochimie. 2013;95(6):1077–86. 10.1016/j.biochi.2012.11.005 . 23178705

[37] Zhang Z, Wang J, He J, Zheng Z, Zeng X, Zhang C, et al Genetic variants in MUC4 gene are associated with lung cancer risk in a Chinese population. PloS one. 2013;8(10):e77723 10.1371/journal.pone.0077723 24204934 PMC3804582

[38] Chang CY, Chang HW, Chen CM, Lin CY, Chen CP, Lai CH, et al MUC4 gene polymorphisms associate with endometriosis development and endometriosis-related infertility. BMC Med. 2011;9:19 10.1186/1741-7015-9-19 21349170 PMC3052195

[39] Xiao X, Wang L, Wei P, Chi Y, Li D, Wang Q, et al Role of MUC20 overexpression as a predictor of recurrence and poor outcome in colorectal cancer. Journal of translational medicine. 2013;11:151 10.1186/1479-5876-11-151 23787019 PMC3702436

[40] Abuli A, Fernandez-Rozadilla C, Alonso-Espinaco V, Munoz J, Gonzalo V, Bessa X, et al Case-control study for colorectal cancer genetic susceptibility in EPICOLON: previously identified variants and mucins. BMC cancer. 2011;11:339 10.1186/1471-2407-11-339 21819567 PMC3176240

[41] Kupcinskas J, Gyvyte U, Bruzaite I, Leja M, Kupcinskaite-Noreikiene R, Pauzas H, et al Common Genetic Variants of PSCA, MUC1 and PLCE1 Genes are not Associated with Colorectal Cancer. Asian Pacific journal of cancer prevention: APJCP. 2015;16(14):6027–32. . 26320491 10.7314/apjcp.2015.16.14.6027

[42] Ahn MH, Bae KB, Kwon JA, Choi HJ, Lee SR, Kim SH, et al Association of MUC6-minisatellite variants with susceptibility to rectal carcinoma. Molecular biology reports. 2013;40(1):303–8. 10.1007/s11033-012-2062-5 . 23054008

[43] Sheng YH, Hasnain SZ, Florin TH, McGuckin MA. Mucins in inflammatory bowel diseases and colorectal cancer. J Gastroenterol Hepatol. 2012;27(1):28–38. 10.1111/j.1440-1746.2011.06909.x . 21913981

[44] Hollingsworth MA, Swanson BJ. Mucins in cancer: protection and control of the cell surface. Nature reviews Cancer. 2004;4(1):45–60. 10.1038/nrc1251 . 14681689

