# Correction: Single nucleotide polymorphisms within MUC4 are associated with colorectal cancer survival

**DOI:** 10.1371/journal.pone.0218064

**Published:** 2019-06-06

**Authors:** Shun Lu, Calogerina Catalano, Stefanie Huhn, Barbara Pardini, Linda Bartu, Veronika Vymetalkova, Ludmila Vodickova, Miroslav Levy, Thomas Buchler, Kari Hemminki, Pavel Vodicka, Asta Försti

The fifth author’s name is spelled incorrectly. The correct name is: Linda Bartu. The correct citation is: Lu S, Catalano C, Huhn S, Pardini B, Bartu L, Vymetalkova V, et al. (2019) Single nucleotide polymorphisms within MUC4 are associated with colorectal cancer survival. PLoS ONE 14(5): e0216666. https://doi.org/10.1371/journal.pone.0216666
