# Supplementary material for: Single nucleotide polymorphisms within MUC4 are associated with colorectal cancer survival
Source: PLoS One. 2019 May 15;14(5):e0216666. doi: 10.1371/journal.pone.0216666 (PMC6519901; doi:10.1371/journal.pone.0216666)
Supplement: S1 Table — (DOCX) [file pone.0216666.s001.docx]

**Single nucleotide polymorphisms within MUC4 are associated with colorectal cancer survival**

Shun Lu^1,2^*, Calogerina Catalano^1^, Stefanie Huhn^1,3^, Barbara Pardini^4^, Linda Partu^5,6^ Veronika Vymetalkova^5,7^, Ludmila Vodickova^5,7.8^, Miroslav Levy^9^, Thomas Buchler^9^, Kari Hemminki^1,10^, Pavel Vodicka^5,7,8^, Asta Försti^1,10^*

^1^Division of Molecular Genetic Epidemiology, German Cancer Research Center, Heidelberg, Germany, ^2^Sichuan Cancer Hospital & Institute, Sichuan Cancer Center, School of Medicine, University of Electronic Science and Technology of China, Chengdu, China. ^3^Department of Multiple Myeloma, Internal Medicine V: Hematology, Oncology and Rheumatology, Heidelberg University Hospital, Heidelberg, Germany, ^4^Italian Institute for Genomic Medicine (IIGM), Turin, Italy, ^5^Department of Molecular Biology of Cancer, Institute of Experimental Medicine, Academy of Sciences of the Czech Republic, Prague, Czech Republic, ^9^Department of Medical Genetics, Third Faculty of Medicine, Charles University, Prague, Czech Republic, ^7^Institute of Biology and Medical Genetics, ^1st^Medical Faculty, Charles University, Prague, Czech Republic, ^8^Biomedical Centre, Faculty of Medicine in Pilsen, Charles University in Prague, Pilsen, Czech Republic, ^9^Department of Oncology, Thomayer Hospital, Prague, Czech Republic, ^10^Center of Primary Health Care Research, Clinical Research Center, Lund University, Malmö, Sweden.

*To whom correspondence should be addressed. Tel: +8602885420897; Fax: +8602885400116; E-mail: [lushun1982@live.cn](mailto:lushun1982@live.cn) ; Tel: +496221421803; Fax: +496221421810; E-mail: [a.foersti@dkfz.de](mailto:a.foersti@dkfz.de)

**S 1** Table. Association of all evaluated SNPs with colorectal cancer susceptibility in the study population of 1532 cases and 1108 controls

| **Gene** | **SNP ID** | **Genotype** | **Risk of CRC adjusted for sex + age** | | | ***P* value** |
| --- | --- | --- | --- | --- | --- | --- |
|  |  |  | **Cases N^a^ (%)** | **Controls N^a^ (%)** | **OR (95%CI)** |  |
| *MUC1* | rs12743084 | C/C | 408 (29.44) | 276 (25.39) | 1 |  |
|  |  | G/C | 653 (47.11) | 517 (47.56) | 1.00 (0.79-1.26) | 1.00 |
|  |  | G/G | 325 (23.45) | 294 (27.05) | 0.82 (0.63-1.07) | 0.15 |
|  |  | G/C+G/G | 978 (70.56) | 811 (74.61) | 0.93 (0.75-1.16) | 0.54 |
|  |  |  |  |  |  |  |
| *MUC1* | rs4072037 | G/G | 413 (28.50) | 263 (25.56) | 1 |  |
|  |  | A/G | 684 (47.20) | 487 (47.33) | 1.02 (0.81-1.29) | 0.86 |
|  |  | A/A | 352 (24.29) | 279 (27.11) | 0.86 (0.66-1.13) | 0.29 |
|  |  | A/G+A/A | 1036 (71.50) | 766 (74.44) | 0.96 (0.77-1.20) | 0.74 |
|  |  |  |  |  |  |  |
| *MUC2* | rs11825977 | G/G | 911 (63.88) | 638 (62.49) | 1 |  |
|  |  | A/G | 457 (32.05) | 335 (32.81) | 0.94 (0.76-1.16) | 0.55 |
|  |  | A/A | 58 (4.07) | 48 (4.70) | 0.86 (0.53-1.41) | 0.55 |
|  |  | A/G+A/A | 515 (36.12) | 383 (37.51) | 0.93 (0.76-1.14) | 0.48 |
|  |  |  |  |  |  |  |
| *MUC2* | rs2071175 | C/C | 1045 (91.43) | 931 (89.52) | 1 |  |
|  |  | C/T | 93 (8.14) | 106 (10.19) | 0.83 (0.58-1.19) | 0.32 |
|  |  | T/T | 5 (0.44) | 3 (0.29) | 1.88 (0.31-11.32) | 0.49 |
|  |  | C/T+ T/T | 98 (8.57) | 109 (10.48) | 0.86 (0.60-1.22) | 0.40 |
|  |  |  |  |  |  |  |
| *MUC2* | rs2856111 | T/T | 1205 (81.31) | 855 (81.27) | 1 |  |
|  |  | C/T | 263 (17.75) | 186 (17.68) | 0.87 (0.68-1.12) | 0.28 |
|  |  | C/C | 14 (0.94) | 11 (1.05) | 1.33 (0.52-3.41) | 0.55 |
|  |  | C/T+ C/C | 277 (18.69) | 197 (18.73) | 0.89 (0.70-1.14) | 0.36 |
|  |  |  |  |  |  |  |
| *MUC5ac* | rs35783651 | G/G | 727 (68.46) | 679 (67.56) | 1 |  |
|  |  | G/C | 306 (28.81) | 298 (29.65) | 1.03 (0.82-1.30) | 0.80 |
|  |  | C/C | 29 (2.73) | 28 (2.79) | 0.77 (0.40-1.51) | 0.45 |
|  |  | G/C+ C/C | 335 (31.54) | 326 (32.44) | 1.01 (0.81-1.26) | 0.95 |
|  |  |  |  |  |  |  |
| *MUC5ac* | rs17859812 | G/G | 916 (64.96) | 685 (64.50) | 1 |  |
|  |  | G/A | 438 (31.06) | 321 (30.23) | 0.99 (0.80-1.23) | 0.93 |
|  |  | A/A | 56 (3.97) | 56 (5.27) | 0.76 (0.48-1.20) | 0.24 |
|  |  | G/A+A/A | 494 (35.04) | 377 (35.50) | 0.96 (0.78-1.17) | 0.66 |
|  |  |  |  |  |  |  |
| *MUC6* | rs11604757 | C/C | 1148 (79.34) | 825 (80.72) | 1 |  |
|  |  | C/T | 271 (18.73) | 179 (17.51) | 1.08 (0.84-1.40) | 0.54 |
|  |  | T/T | 28 (1.94) | 18 (1.76) | 1.56 (0.76-3.20) | 0.23 |
|  |  | C/T+ T/T | 299 (20.66) | 197 (19.28) | 1.12 (0.88-1.43) | 0.36 |
|  |  |  |  |  |  |  |
| *MUC6* | rs61869016 | A/A | 622 (44.08) | 408 (41.85) | 1 |  |
|  |  | G/A | 624 (44.22) | 470 (48.21) | **0.74 (0.60-0.92)** | **0.01** |
|  |  | G/G | 165 (11.69) | 97 (9.95) | 0.99 (0.70-1.38) | 0.94 |
|  |  | G/A+G/G | 789 (55.92) | 567 (58.15) | **0.78 (0.64-0.96)** | **0.02** |
|  |  |  |  |  |  |  |
| *MUC6* | rs6597947 | C/C | 1148 (78.95) | 863 (80.73) | 1 |  |
|  |  | A/C | 289 (19.88) | 189 (17.68) | 1.13 (0.88-1.45) | 0.33 |
|  |  | A/A | 17 (1.17) | 17 (1.59) | 0.86 (0.38-1.97) | 0.72 |
|  |  | A/C+A/A | 306 (21.05) | 206 (19.27) | 1.11 (0.87-1.41) | 0.39 |
|  |  |  |  |  |  |  |
| *MUC6* | rs72842418 | T/T | 1106 (75.29) | 789 (74.22) | 1 |  |
|  |  | T/C | 360 (24.51) | 274 (25.78) | 0.93 (0.75-1.17) | 0.55 |
|  |  | C/C | 3 (0.20) | 0 (0.00) | - | - |
|  |  | T/C+ C/C | 363 (24.71) | 274 (25.78) | 0.94 (0.75-1.18) | 0.59 |
|  |  |  |  |  |  |  |
| *MUC6* | rs7396383 | T/T | 810 (57.20) | 591 (59.16) | 1 |  |
|  |  | A/T | 519 (36.65) | 339 (33.93) | 1.17 (0.94-1.44) | 0.15 |
|  |  | A/A | 87 (6.14) | 69 (6.91) | 0.98 (0.66-1.47) | 0.93 |
|  |  | A/T+A/A | 606 (42.80) | 408 (40.84) | 1.14 (0.93-1.39) | 0.21 |
|  |  |  |  |  |  |  |
| *MUC6* | rs7481521 | T/T | 411 (28.92) | 295 (30.41) | 1 |  |
|  |  | C/T | 696 (48.98) | 456 (47.01) | 1.09 (0.87-1.37) | 0.46 |
|  |  | C/C | 314 (22.10) | 219 (22.58) | 1.10 (0.83-1.45) | 0.50 |
|  |  | C/T+ C/C | 1010 (71.08) | 675 (69.59) | 1.09 (0.88-1.36) | 0.42 |
|  |  |  |  |  |  |  |
| *B3GNT6* | rs12271271 | G/G | 875 (61.66) | 663 (60.99) | 1 |  |
|  |  | G/A | 477 (33.62) | 376 (34.59) | 0.93 (0.75-1.14) | 0.49 |
|  |  | A/A | 67 (4.72) | 48 (4.42) | 1.04 (0.64-1.68) | 0.87 |
|  |  | G/A + A/A | 544 (38.34) | 424 (39.01) | 0.94 (0.77-1.15) | 0.55 |
|  |  |  |  |  |  |  |
| *B3GNT6* | rs12422079 | A/A | 739 (53.78) | 480 (51.95) | 1 |  |
|  |  | A/C | 526 (38.28) | 383 (41.45) | 0.94 (0.76-1.16) | 0.56 |
|  |  | C/C | 109 (7.93) | 61 (6.60) | 1.20 (0.79-1.82) | 0.39 |
|  |  | A/C+C/C | 635 (46.22) | 444 (48.05) | 0.97 (0.79-1.20) | 0.79 |
|  |  |  |  |  |  |  |
| *B3GNT6* | rs58116088 | G/G | 554 (37.61) | 393 (37.68) | 1 |  |
|  |  | G/A | 709 (48.13) | 527 (50.53) | 0.92 (0.74-1.13) | 0.43 |
|  |  | A/A | 210 (14.26) | 123 (11.79) | 1.15 (0.83-1.58) | 0.40 |
|  |  | G/A+A/A | 919 (62.39) | 650 (62.32) | 0.96 (0.78-1.18) | 0.70 |
|  |  |  |  |  |  |  |
| *B3GNT6* | rs61902094 | G/G | 1014 (70.37) | 749 (70.26) | 1 |  |
|  |  | G/A | 389 (27.00) | 293 (27.49) | 1.00 (0.81-1.25) | 0.97 |
|  |  | A/A | 38 (2.64) | 24 (2.25) | 1.22 (0.65-2.30) | 0.53 |
|  |  | G/A+A/A | 427 (29.63) | 317 (29.74) | 1.02 (0.83-1.26) | 0.85 |
|  |  |  |  |  |  |  |
| *B3GNT6* | rs6592699 | G/G | 759 (52.24) | 556 (51.06) | 1 |  |
|  |  | A/G | 585 (40.26) | 452 (41.51) | 0.97 (0.80-1.19) | 0.80 |
|  |  | A/A | 109 (7.50) | 81 (7.44) | 0.98 (0.68-1.42) | 0.93 |
|  |  | A/G+A/A | 694 (47.76) | 533 (48.94) | 0.98 (0.81-1.18) | 0.80 |
|  |  |  |  |  |  |  |
| *B3GNT6* | rs73493606 | C/C | 1226 (84.61) | 908 (85.42) | 1 |  |
|  |  | C/T | 213 (14.70) | 147 (13.83) | 0.99 (0.75-1.31) | 0.94 |
|  |  | T/T | 10 (0.69) | 8 (0.75) | 1.02 (0.31-3.37) | 0.98 |
|  |  | C/T+ T/T | 223 (15.39) | 155 (14.58) | 0.99 (0.75-1.30) | 0.95 |
|  |  |  |  |  |  |  |
| *MUC4* | rs3749331 | C/C | 797 (73.39) | 677 (70.16) | 1 |  |
|  |  | C/T | 255 (23.48) | 259 (26.84) | 0.83 (0.65-1.07) | 0.15 |
|  |  | T/T | 34 (3.13) | 29 (3.01) | 1.01 (0.55-1.85) | 0.98 |
|  |  | C/T+ T/T | 289 (26.61) | 288 (29.84) | 0.85 (0.67-1.08) | 0.18 |
|  |  |  |  |  |  |  |
| *MUC4* | rs3107764 | G/G | 487 (36.98) | 354 (39.86) | 1 |  |
|  |  | C/G | 624 (47.38) | 404 (45.50) | 1.06 (0.85-1.33) | 0.60 |
|  |  | C/C | 206 (15.64) | 130 (14.64) | 1.17 (0.85-1.60) | 0.33 |
|  |  | C/G+ C/C | 830 (63.02) | 534 (60.14) | 1.09 (0.88-1.35) | 0.44 |
|  |  |  |  |  |  |  |
| *MUC4* | rs2246901 | A/A | 727 (51.34) | 527 (53.39) | 1 |  |
|  |  | A/C | 577 (40.75) | 373 (37.79) | 1.13 (0.91-1.39) | 0.27 |
|  |  | C/C | 112 (7.91) | 87 (8.81) | 0.97 (0.67-1.41) | 0.88 |
|  |  | A/C+ C/C | 689 (48.66) | 460 (46.61) | 1.10 (0.90-1.34) | 0.36 |
|  |  |  |  |  |  |  |
| *MUC4* | rs842225 | G/G | 407 (28.58) | 265 (26.85) | 1 |  |
|  |  | A/G | 694 (48.74) | 486 (49.24) | 0.95 (0.75-1.20) | 0.64 |
|  |  | A/A | 323 (22.68) | 236 (23.91) | 0.85 (0.65-1.13) | 0.27 |
|  |  | A/G+A/A | 1017 (71.42) | 722 (73.15) | 0.92 (0.73-1.14) | 0.44 |

^a^Number of cases may differ due to missing data

N number of subjects, OR odds ratio, CI confidence interval. Bold numbers indicate a statistical significance at 5% lev
