# Supplementary material for: Single nucleotide polymorphisms within MUC4 are associated with colorectal cancer survival
Source: PLoS One. 2019 May 15;14(5):e0216666. doi: 10.1371/journal.pone.0216666 (PMC6519901; doi:10.1371/journal.pone.0216666)
Supplement: S2 Table — (DOCX) [file pone.0216666.s002.docx]

**S2 Table.** Association of all evaluated SNPs with overall survival of all colorectal cancer patients (n=672) and overall and event-free survival among patients without distant metastasis at the time of diagnosis (M=0, n=494)

| **Gene** | **SNP ID** | **Genotype** | **Overall Survival Analysis (adjusted for age, sex and stage)** | | |  | **Overal Survival Analysis (M=0) adjusted for age, sex and stage** | | |  | **Event Free Analysis (M=0) adjusted for age, sex and stage** | | |  |
| --- | --- | --- | --- | --- | --- | --- | --- | --- | --- | --- | --- | --- | --- | --- |
|  |  |  | **N^a^** | **N^a^ died (%)** | **HR (95%CI)** | ***P* value** | **N^a^** | **N^a^ died (%)** | **HR (95%CI)** | ***P* value** | **N^a^** | **N^a^ died (%)** | **HR (95%CI)** | ***P* value** |
| *MUC1* | rs12743084 | C/C | 175 | 74 (42.29) | 1 |  | 126 | 39 (30.95) | 1 |  | 126 | 45 (35.71) | 1 |  |
|  |  | G/C | 293 | 127 (43.34) | 1.27 (0.95-1.69) | 0.11 | 227 | 70 (30.84) | 1.19 (0.80-1.77) | 0.38 | 227 | 75 (33.04) | 1.07 (0.74-1.56) | 0.73 |
|  |  | G/G | 142 | 66 (46.48) | 1.33 (0.95-1.85) | 0.10 | 100 | 32 (32.00) | 1.14 (0.71-1.83) | 0.59 | 100 | 37 (37.00) | 1.20 (0.77-1.87) | 0.43 |
|  |  | G/C+G/G | 435 | 193 (44.37) | 1.29 (0.98-1.69) | 0.07 | 327 | 102 (31.19) | 1.18 (0.81-1.71) | 0.39 | 327 | 112 (34.25) | 1.11 (0.78-1.57) | 0.57 |
|  |  |  |  |  |  |  |  |  |  |  |  |  |  |  |
| *MUC1* | rs4072037 | G/G | 173 | 76 (43.93) | 1 |  | 128 | 45 (35.16) | 1 |  | 128 | 50 (39.06) | 1 |  |
|  |  | A/G | 310 | 136 (43.87) | 1.12 (0.85-1.49) | 0.42 | 234 | 68 (29.06) | 0.96 (0.66-1.41) | 0.85 | 234 | 74 (31.62) | 0.89 (0.62-1.27) | 0.51 |
|  |  | A/A | 153 | 74 (48.37) | 1.25 (0.90-1.72) | 0.18 | 110 | 38 (34.55) | 1.03 (0.66-1.59) | 0.91 | 110 | 43 (39.09) | 1.11 (0.74-1.69) | 0.61 |
|  |  | A/G+A/A | 463 | 210 (45.36) | 1.16 (0.89-1.51) | 0.26 | 344 | 106 (30.81) | 0.99 (0.69-1.40) | 0.94 | 344 | 117 (34.01) | 0.96 (0.68-1.34) | 0.79 |
|  |  |  |  |  |  |  |  |  |  |  |  |  |  |  |
| *MUC2* | rs11825977 | G/G | 418 | 190 (45.45) | 1 |  | 309 | 98 (31.72) | 1 |  | 309 | 108 (34.95) | 1 |  |
|  |  | A/G | 185 | 78 (42.16) | 0.98 (0.75-1.27) | 0.86 | 135 | 39 (28.89) | 0.97 (0.67-1.41) | 0.88 | 135 | 43 (31.85) | 0.99 (0.69-1.41) | 0.95 |
|  |  | A/A | 24 | 11 (45.83) | 1.17 (0.64-2.16) | 0.61 | 17 | 4 (23.53) | 0.88 (0.32-2.41) | 0.81 | 17 | 5 (29.41) | 0.80 (0.33-1.97) | 0.63 |
|  |  | A/G+A/A | 209 | 89 (42.58) | 1.00 (0.77-1.28) | 0.98 | 152 | 43 (28.29) | 0.96 (0.67-1.38) | 0.84 | 152 | 48 (31.58) | 0.97 (0.69-1.36) | 0.84 |
|  |  |  |  |  |  |  |  |  |  |  |  |  |  |  |
| *MUC2* | rs2071175 | C/C | 513 | 221 (43.08) | 1 |  | 382 | 115 (30.10) | 1 |  | 382 | 127 (33.25) | 1 |  |
|  |  | C/T | 38 | 20 (52.63) | 0.94 (0.59-1.50) | 0.81 | 26 | 11 (42.31) | 1.47 (0.79-2.76) | 0.23 | 26 | 11 (42.31) | 1.17 (0.63-2.17) | 0.63 |
|  |  | T/T | 4 | 2 (50.00) | **4.47 (1.09-18.30)** | **0.04** | 3 | 1 (33.33) | 4.14 (0.56-30.46) | 0.16 | 3 | 1 (33.33) | 2.46 (0.34-17.91) | 0.37 |
|  |  | C/T+ T/T | 42 | 22 (52.38) | 1.02 (0.66-1.59) | 0.93 | 29 | 12 (41.38) | 1.56 (0.85-2.85) | 0.15 | 29 | 12 (41.38) | 1.22 (0.67-2.22) | 0.51 |
|  |  |  |  |  |  |  |  |  |  |  |  |  |  |  |
| *MUC2* | rs2856111 | T/T | 520 | 234 (45.00) | 1 |  | 375 | 116 (30.93) | 1 |  | 375 | 130 (34.67) | 1 |  |
|  |  | C/T | 115 | 50 (43.48) | 0.89 (0.66-1.21) | 0.47 | 91 | 31 (34.07) | 1.00 (0.67-1.50) | 0.98 | 91 | 34 (37.36) | 1.08 (0.74-1.58) | 0.70 |
|  |  | C/C | 12 | 8 (66.67) | **2.55 (1.25-5.21)** | **0.01** | 7 | 3 (42.86) | 3.04 (0.94-9.82) | 0.06 | 7 | 3 (42.86) | 1.84 (0.58-5.89) | 0.30 |
|  |  | C/T+ C/C | 127 | 58 (45.67) | 0.98 (0.73-1.31) | 0.89 | 98 | 34 (34.69) | 1.07 (0.72-1.57) | 0.75 | 98 | 37 (37.76) | 1.11 (0.77-1.61) | 0.57 |
|  |  |  |  |  |  |  |  |  |  |  |  |  |  |  |
| *MUC4* | rs3749331 | C/C | 356 | 147 (41.29) | 1 |  | 277 | 85 (30.69) | 1 |  | 277 | 92 (33.21) | 1 |  |
|  |  | C/T | 87 | 40 (45.98) | 0.81 (0.57-1.15) | 0.24 | 62 | 22 (35.48) | 1.12 (0.70-1.80) | 0.64 | 62 | 27 (43.55) | 1.31 (0.85-2.02) | 0.22 |
|  |  | T/T | 15 | 7 (46.67) | 1.28 (0.59-2.75) | 0.53 | 10 | 3 (30.00) | 0.91 (0.29-2.92) | 0.88 | 10 | 4 (40.00) | 1.16 (0.42-3.17) | 0.78 |
|  |  | C/T+ T/T | 102 | 47 (46.08) | 0.86 (0.61-1.20) | 0.37 | 72 | 25 (34.72) | 1.09 (0.69-1.71) | 0.71 | 72 | 31 (43.06) | 1.29 (0.85-1.94) | 0.23 |
|  |  |  |  |  |  |  |  |  |  |  |  |  |  |  |
| *MUC4* | rs3107764 | G/G | 199 | 84 (42.21) | 1 |  | 152 | 40 (26.32) | 1 |  | 152 | 45 (29.61) | 1 |  |
|  |  | C/G | 283 | 119 (42.05) | 0.97 (0.73-1.29) | 0.84 | 215 | 65 (30.23) | 1.13 (0.76-1.69) | 0.54 | 215 | 75 (34.88) | 1.14 (0.79-1.66) | 0.48 |
|  |  | C/C | 86 | 40 (46.51) | 1.06 (0.72-1.55) | 0.77 | 66 | 26 (39.39) | **2.08 (1.26-3.43)** | **0.00** | 66 | 28 (42.42) | **1.79 (1.12-2.88)** | **0.02** |
|  |  | C/G+ C/C | 369 | 159 (43.09) | 0.99 (0.76-1.29) | 0.95 | 281 | 91 (32.38) | 1.31 (0.90-1.91) | 0.16 | 281 | 103 (36.65) | 1.27 (0.89-1.82) | 0.18 |
|  |  |  |  |  |  |  |  |  |  |  |  |  |  |  |
| *MUC4* | rs2246901 | A/A | 311 | 137 (44.05) | 1 |  | 225 | 64 (28.44) | 1 |  | 225 | 71 (31.56) | 1 |  |
|  |  | A/C | 262 | 116 (44.27) | 1.07 (0.84-1.38) | 0.57 | 193 | 62 (32.12) | 1.25 (0.88-1.77) | 0.21 | 193 | 68 (35.23) | 1.24 (0.89-1.73) | 0.21 |
|  |  | C/C | 49 | 23 (46.94) | 0.98 (0.63-1.54) | 0.94 | 41 | 17 (41.46) | 1.47 (0.86-2.53) | 0.16 | 41 | 17 (41.46) | 1.33 (0.78-2.28) | 0.29 |
|  |  | A/C+ C/C | 311 | 139 (44.69) | 1.06 (0.84-1.34) | 0.64 | 234 | 79 (33.76) | 1.29 (0.93-1.80) | 0.13 | 234 | 85 (36.32) | 1.26 (0.92-1.72) | 0.16 |
|  |  |  |  |  |  |  |  |  |  |  |  |  |  |  |
| *MUC4* | rs842225 | G/G | 176 | 79 (44.89) | 1 |  | 135 | 45 (33.33) | 1 |  | 135 | 50 (37.04) | 1 |  |
|  |  | A/G | 295 | 131 (44.41) | 1.06 (0.80-1.40) | 0.70 | 216 | 69 (31.94) | 0.92 (0.63-1.34) | 0.66 | 216 | 77 (35.65) | 0.96 (0.68-1.38) | 0.84 |
|  |  | A/A | 144 | 63 (43.75) | 0.96 (0.69-1.34) | 0.83 | 103 | 26 (25.24) | **0.61 (0.37-0.99)** | **0.05** | 103 | 28 (27.18) | **0.62 (0.39-0.99)** | **0.05** |
|  |  | A/G+A/A | 439 | 194 (44.19) | 1.03 (0.79-1.33) | 0.85 | 319 | 95 (29.78) | 0.81 (0.57-1.15) | 0.24 | 319 | 105 (32.92) | 0.84 (0.60-1.18) | 0.32 |
|  |  |  |  |  |  |  |  |  |  |  |  |  |  |  |
| *MUC5ac* | rs35783651 | G/G | 274 | 137 (50.00) | 1 |  | 199 | 73 (36.68) | 1 |  | 199 | 79 (39.70) | 1 |  |
|  |  | G/C | 114 | 48 (42.11) | 0.73 (0.52-1.01) | 0.06 | 78 | 23 (29.49) | 0.80 (0.49-1.29) | 0.36 | 78 | 26 (33.33) | 0.80 (0.51-1.26) | 0.34 |
|  |  | C/C | 12 | 6 (50.00) | 1.32 (0.57-3.02) | 0.51 | 9 | 3 (33.33) | 0.87 (0.27-2.81) | 0.82 | 9 | 4 (44.44) | 1.33 (0.48-3.70) | 0.58 |
|  |  | G/C+ C/C | 126 | 54 (42.86) | 0.77 (0.56-1.05) | 0.10 | 87 | 26 (29.89) | 0.81 (0.51-1.28) | 0.36 | 87 | 30 (34.48) | 0.85 (0.55-1.31) | 0.45 |
|  |  |  |  |  |  |  |  |  |  |  |  |  |  |  |
| *MUC5ac* | rs17859812 | G/G | 405 | 187 (46.17) | 1 |  | 291 | 89 (30.58) | 1 |  | 291 | 99 (34.02) | 1 |  |
|  |  | G/A | 197 | 90 (45.69) | 0.97 (0.75-1.25) | 0.81 | 152 | 53 (34.87) | 1.14 (0.81-1.61) | 0.46 | 152 | 60 (39.47) | 1.14 (0.82-1.57) | 0.44 |
|  |  | A/A | 25 | 9 (36.00) | 1.01 (0.51-1.99) | 0.97 | 20 | 5 (25.00) | 1.04 (0.42-2.56) | 0.94 | 20 | 5 (25.00) | 0.85 (0.34-2.09) | 0.72 |
|  |  | G/A+A/A | 222 | 99 (44.59) | 0.97 (0.76-1.24) | 0.82 | 172 | 58 (33.72) | 1.13 (0.81-1.58) | 0.47 | 172 | 65 (37.79) | 1.11 (0.81-1.51) | 0.53 |
|  |  |  |  |  |  |  |  |  |  |  |  |  |  |  |
| *MUC6* | rs11604757 | C/C | 503 | 230 (45.73) | 1 |  | 368 | 116 (31.52) | 1 |  | 368 | 131 (35.60) | 1 |  |
|  |  | C/T | 118 | 52 (44.07) | 1.08 (0.80-1.46) | 0.61 | 88 | 31 (35.23) | 1.39 (0.93-2.08) | 0.11 | 88 | 32 (36.36) | 1.18 (0.80-1.74) | 0.42 |
|  |  | T/T | 12 | 6 (50.00) | 0.86 (0.38-1.93) | 0.71 | 7 | 2 (28.57) | 1.13 (0.27-4.61) | 0.87 | 7 | 2 (28.57) | 0.86 (0.21-3.51) | 0.84 |
|  |  | C/T+ T/T | 130 | 58 (44.62) | 1.05 (0.79-1.41) | 0.73 | 95 | 33 (34.74) | 1.37 (0.93-2.03) | 0.11 | 95 | 34 (35.79) | 1.15 (0.79-1.68) | 0.47 |
|  |  |  |  |  |  |  |  |  |  |  |  |  |  |  |
| *MUC6* | rs61869016 | A/A | 279 | 119 (42.65) | 1 |  | 217 | 73 (33.64) | 1 |  | 217 | 78 (35.94) | 1 |  |
|  |  | G/A | 278 | 122 (43.88) | 0.86 (0.67-1.11) | 0.24 | 197 | 53 (26.90) | **0.65 (0.46-0.93)** | **0.02** | 197 | 62 (31.47) | 0.77 (0.55-1.08) | 0.13 |
|  |  | G/G | 60 | 31 (51.67) | 1.02 (0.68-1.51) | 0.93 | 40 | 14 (35.00) | 0.85 (0.48-1.52) | 0.59 | 40 | 15 (37.50) | 0.93 (0.54-1.63) | 0.81 |
|  |  | G/A+G/G | 338 | 153 (45.27) | 0.89 (0.70-1.13) | 0.33 | 237 | 67 (28.27) | **0.68 (0.49-0.96)** | **0.03** | 237 | 77 (32.49) | 0.80 (0.58-1.10) | 0.17 |
|  |  |  |  |  |  |  |  |  |  |  |  |  |  |  |
| *MUC6* | rs6597947 | C/C | 506 | 225 (44.47) | 1 |  | 373 | 118 (31.64) | 1 |  | 373 | 130 (34.85) | 1 |  |
|  |  | A/C | 130 | 59 (45.38) | 1.10 (0.82-1.46) | 0.53 | 93 | 28 (30.11) | 0.93 (0.62-1.41) | 0.75 | 93 | 32 (34.41) | 1.00 (0.68-1.47) | 0.99 |
|  |  | A/A | 7 | 2 (28.57) | 2.15 (0.53-8.80) | 0.29 | 7 | 2 (28.57) | 2.17 (0.53-8.89) | 0.28 | 7 | 2 (28.57) | 1.79 (0.44-7.31) | 0.42 |
|  |  | A/C+A/A | 137 | 61 (44.53) | 1.12 (0.84-1.48) | 0.45 | 100 | 30 (30.00) | 0.97 (0.65-1.46) | 0.89 | 100 | 34 (34.00) | 1.02 (0.70-1.50) | 0.90 |
|  |  |  |  |  |  |  |  |  |  |  |  |  |  |  |
| *MUC6* | rs72842418 | T/T | 477 | 217 (45.49) | 1 |  | 343 | 108 (31.49) | 1 |  | 343 | 122 (35.57) | 1 |  |
|  |  | T/C | 162 | 69 (42.59) | 1.05 (0.80-1.38) | 0.71 | 128 | 40 (31.25) | 1.00 (0.69-1.45) | 1.00 | 128 | 41 (32.03) | 0.82 (0.57-1.17) | 0.28 |
|  |  | C/C | 2 | 0 (0.00) | - | - | 2 | 0 (0.00) | - | - | 2 | 0 (0.00) | - | - |
|  |  | T/C+ C/C | 164 | 69 (42.07) | 1.05 (0.80-1.38) | 0.73 | 130 | 40 (30.77) | 1.00 (0.69-1.44) | 0.99 | 130 | 41 (31.54) | 0.81 (0.57-1.16) | 0.26 |
|  |  |  |  |  |  |  |  |  |  |  |  |  |  |  |
| *MUC6* | rs7396383 | T/T | 347 | 155 (44.67) | 1 |  | 257 | 80 (31.13) | 1 |  | 257 | 89 (34.63) | 1 |  |
|  |  | A/T | 232 | 103 (44.40) | 0.99 (0.77-1.28) | 0.96 | 170 | 51 (30.00) | 1.01 (0.71-1.44) | 0.96 | 170 | 57 (33.53) | 1.03 (0.74-1.44) | 0.87 |
|  |  | A/A | 39 | 14 (35.90) | 1.06 (0.61-1.84) | 0.83 | 30 | 9 (30.00) | 1.08 (0.54-2.17) | 0.82 | 30 | 10 (33.33) | 1.09 (0.56-2.10) | 0.80 |
|  |  | A/T+A/A | 271 | 117 (43.17) | 1.00 (0.79-1.27) | 0.99 | 200 | 60 (30.00) | 1.02 (0.73-1.43) | 0.92 | 200 | 67 (33.50) | 1.04 (0.75-1.43) | 0.82 |
|  |  |  |  |  |  |  |  |  |  |  |  |  |  |  |
| *MUC6* | rs7481521 | T/T | 161 | 74 (45.96) | 1 |  | 115 | 37 (32.17) | 1 |  | 115 | 43 (37.39) | 1 |  |
|  |  | C/T | 306 | 132 (43.14) | 1.01 (0.76-1.35) | 0.94 | 230 | 70 (30.43) | 0.95 (0.64-1.42) | 0.81 | 230 | 76 (33.04) | 0.85 (0.58-1.24) | 0.40 |
|  |  | C/C | 137 | 56 (40.88) | 0.97 (0.68-1.37) | 0.85 | 110 | 36 (32.73) | 1.15 (0.73-1.83) | 0.55 | 110 | 40 (36.36) | 1.00 (0.65-1.55) | 0.98 |
|  |  | C/T+ C/C | 443 | 188 (42.44) | 1.00 (0.76-1.31) | 0.99 | 340 | 106 (31.18) | 1.01 (0.69-1.47) | 0.95 | 340 | 116 (34.12) | 0.90 (0.63-1.28) | 0.55 |
|  |  |  |  |  |  |  |  |  |  |  |  |  |  |  |
| *B3GNT6* | rs12271271 | G/G | 381 | 161 (42.26) | 1 |  | 281 | 82 (29.18) | 1 |  | 281 | 93 (33.10) | 1 |  |
|  |  | G/A | 206 | 98 (47.57) | 1.09 (0.85-1.41) | 0.49 | 152 | 54 (35.53) | 1.07 (0.76-1.52) | 0.69 | 152 | 57 (37.50) | 1.06 (0.76-1.47) | 0.75 |
|  |  | A/A | 35 | 13 (37.14) | 0.93 (0.53-1.64) | 0.80 | 26 | 5 (19.23) | 0.64 (0.26-1.59) | 0.34 | 26 | 5 (19.23) | 0.51 (0.21-1.26) | 0.15 |
|  |  | G/A + A/A | 241 | 111 (46.06) | 1.07 (0.84-1.37) | 0.58 | 178 | 59 (33.15) | 1.02 (0.72-1.42) | 0.93 | 178 | 62 (34.83) | 0.97 (0.70-1.35) | 0.87 |
|  |  |  |  |  |  |  |  |  |  |  |  |  |  |  |
| *B3GNT6* | rs12422079 | A/A | 317 | 137 (43.22) | 1 |  | 234 | 71 (30.34) | 1 |  | 234 | 83 (35.47) | 1 |  |
|  |  | A/C | 230 | 101 (43.91) | 0.97 (0.75-1.26) | 0.84 | 173 | 51 (29.48) | 0.78 (0.54-1.12) | 0.18 | 173 | 54 (31.21) | **0.70 (0.49-0.98)** | **0.04** |
|  |  | C/C | 48 | 25 (52.08) | 1.33 (0.86-2.04) | 0.20 | 30 | 12 (40.00) | 1.62 (0.87-3.01) | 0.13 | 30 | 12 (40.00) | 1.24 (0.67-2.28) | 0.49 |
|  |  | A/C+C/C | 278 | 126 (45.32) | 1.03 (0.81-1.31) | 0.82 | 203 | 63 (31.03) | 0.87 (0.62-1.22) | 0.42 | 203 | 66 (32.51) | 0.76 (0.55-1.05) | 0.10 |
|  |  |  |  |  |  |  |  |  |  |  |  |  |  |  |
| *B3GNT6* | rs58116088 | G/G | 239 | 115 (48.12) | 1 |  | 172 | 58 (33.72) | 1 |  | 172 | 65 (37.79) | 1 |  |
|  |  | G/A | 318 | 130 (40.88) | 0.79 (0.62-1.02) | 0.07 | 242 | 72 (29.75) | 0.77 (0.54-1.09) | 0.14 | 242 | 78 (32.23) | 0.78 (0.56-1.09) | 0.14 |
|  |  | A/A | 87 | 42 (48.28) | 0.75 (0.53-1.07) | 0.11 | 61 | 19 (31.15) | 0.75 (0.45-1.27) | 0.29 | 61 | 22 (36.07) | 0.87 (0.54-1.41) | 0.57 |
|  |  | G/A+A/A | 405 | 172 (42.47) | **0.78 (0.62-0.99)** | **0.04** | 303 | 91 (30.03) | 0.76 (0.55-1.07) | 0.11 | 303 | 100 (33.00) | 0.80 (0.58-1.09) | 0.16 |
|  |  |  |  |  |  |  |  |  |  |  |  |  |  |  |
| *B3GNT6* | rs61902094 | G/G | 436 | 195 (44.72) | 1 |  | 322 | 101 (31.37) | 1 |  | 322 | 115 (35.71) | 1 |  |
|  |  | G/A | 180 | 83 (46.11) | 0.96 (0.74-1.24) | 0.74 | 131 | 42 (32.06) | 0.88 (0.61-1.26) | 0.49 | 131 | 43 (32.82) | 0.81 (0.57-1.15) | 0.23 |
|  |  | A/A | 18 | 9 (50.00) | 1.01 (0.52-1.99) | 0.97 | 12 | 4 (33.33) | 0.98 (0.36-2.69) | 0.97 | 12 | 4 (33.33) | 0.86 (0.32-2.36) | 0.77 |
|  |  | G/A+A/A | 198 | 92 (46.46) | 0.96 (0.75-1.24) | 0.77 | 143 | 46 (32.17) | 0.89 (0.62-1.26) | 0.50 | 143 | 47 (32.87) | 0.81 (0.58-1.14) | 0.23 |
|  |  |  |  |  |  |  |  |  |  |  |  |  |  |  |
| *B3GNT6* | rs6592699 | G/G | 324 | 146 (45.06) | 1 |  | 234 | 71 (30.34) | 1 |  | 234 | 78 (33.33) | 1 |  |
|  |  | A/G | 256 | 115 (44.92) | 1.17 (0.92-1.50) | 0.21 | 193 | 65 (33.68) | 1.34 (0.96-1.89) | 0.09 | 193 | 72 (37.31) | 1.26 (0.92-1.75) | 0.16 |
|  |  | A/A | 48 | 22 (45.83) | 1.14 (0.73-1.78) | 0.58 | 33 | 9 (27.27) | 0.99 (0.50-1.99) | 0.99 | 33 | 12 (36.36) | 1.12 (0.61-2.06) | 0.72 |
|  |  | A/G+A/A | 304 | 137 (45.07) | 1.17 (0.92-1.47) | 0.20 | 226 | 74 (32.74) | 1.29 (0.93-1.79) | 0.13 | 226 | 84 (37.17) | 1.24 (0.91-1.69) | 0.17 |
|  |  |  |  |  |  |  |  |  |  |  |  |  |  |  |
| *B3GNT6* | rs73493606 | C/C | 530 | 233 (43.96) | 1 |  | 390 | 120 (30.77) | 1 |  | 390 | 133 (34.10) | 1 |  |
|  |  | C/T | 91 | 40 (43.96) | 1.04 (0.74-1.46) | 0.80 | 73 | 26 (35.62) | 1.34 (0.88-2.06) | 0.18 | 73 | 27 (36.99) | 1.20 (0.79-1.82) | 0.39 |
|  |  | T/T | 6 | 1 (16.67) | 0.50 (0.07-3.60) | 0.49 | 5 | 0 (0.00) | - | - | 5 | 2 (40.00) | 1.54 (0.38-6.27) | 0.55 |
|  |  | C/T+ T/T | 97 | 41 (42.27) | 1.02 (0.73-1.42) | 0.92 | 78 | 26 (33.33) | 1.27 (0.82-1.94) | 0.28 | 78 | 29 (37.18) | 1.22 (0.81-1.83) | 0.34 |
|  |  |  |  |  |  |  |  |  |  |  |  |  |  |  |

^a^Number of cases may differ due to missing data

N number of subjects, OR odds ratio, CI confidence interval. Bold numbers indicate a statistical significance at 5% level
